# Stem-Cell-Assisted Lipotransfer and Platelet-Rich Plasma in Breast Reconstruction: A Literature Review

**DOI:** 10.1007/s00266-025-04921-w

**Published:** 2025-05-21

**Authors:** Aikaterini-Gavriela Giannakaki, Aikaterini Stylianaki, Maria-Nektaria Giannakaki, Sophia Koura, Eutychia Papachatzopoulou, Ioannis Papapanagiotou, Dimitris Baroutis, Dionysia Rompoti, Spyros Marinopoulos, Eleni-Sivylla Bikouvaraki, Dimitriοs Karathanasis, Dimitriοs Pappas, Kalliopi Pappa, Georgios Daskalakis, Constantine Dimitrakakis

**Affiliations:** 1 First Department of Obstetrics and Gynecology, Alexandra University Hospital, National and Kapodistrian University of Athens, 11528 Athens, Greece; 2 Medical School, Aristotle University of Thessaloniki, 54124 Thessaloniki, Greece; 3 Laboratory of Cell and Gene Therapy, Biomedical Research Foundation of the Academy of Athens, Athens, Greece; 4 Allegene PHARMALABS GmbH, 1020 Vienna, Austria

**Keywords:** Autologous fat graft, Breast, Cell-assisted lipotransfer, Adipose tissue-derived stem cells, Platelet-rich plasma

## Abstract

**Background:**

Autologous fat transplantation to the breast, a minimal invasive technique, has significantly expanded in aesthetic and reconstructive surgery over the past two decades. Initially used for lumpectomy defects or correcting contour deformities post-breast reconstruction, it is now also performed as a stand-alone technique for primary breast augmentation or as a complementary method to implant-based augmentation. However, this surgical technique is associated with a high absorption rate. The use of fat grafts for breast augmentation remains controversial due to concerns about its safety, efficacy, and impact on breast cancer.

**Methods:**

This review examines the literature on cell-assisted lipotransfer (CAL) and PRP-assisted lipotransfer, comparing fat graft survival, complication rates, and oncological safety with conventional autologous lipotransfer.

**Results:**

CAL and PRP-assisted lipotransfer techniques demonstrate improved fat graft retention and reduced complications compared to conventional methods. Several studies report a potential reduction in the absorption rate of fat grafts with improvements in aesthetic outcomes.

**Conclusions:**

While CAL and PRP-assisted lipotransfer have shown promising results in enhancing fat graft survival and reducing complications, there are still concerns about their oncological safety.

**No Level Assigned:**

This journal requires that authors assign a level of evidence to each submission to which Evidence-Based Medicine rankings are applicable. This excludes Review Articles, Book Reviews, and manuscripts that concern Basic Science, Animal Studies, Cadaver Studies, and Experimental Studies. For a full description of these Evidence-Based Medicine ratings, please refer to the Table of Contents or the online Instructions to Authors www.springer.com/00266.

## Introduction

Autologous fat transplantation is a popular technique for repairing volume defects from breast cancer surgery, breastfeeding, aging, and several congenital anomalies [1]. Its popularity in plastic surgery is due to its minimally invasive nature for breast regeneration and reconstruction. Currently, it serves as an alternative technique for breast reconstruction, mitigating postoperative complications and risks associated with breast implants, such as anaplastic large-cell lymphoma [2]. Autologous fat graft is biocompatible, non-immunogenic, and easily available and readily available, making it suitable for contour deformities [3]. However, autologous fat transfer has a low graft survival rate, which ranges from 25 to 80% [4, 5].

Recently, enhancing autologous fat grafts with stromal vascular fraction (SVF) has been proposed to improve viability. SVF includes adipose-derived mesenchymal stromal cells (ASCs), endothelial cells (ECs), endothelial progenitor cells (EPCs), pericytes, preadipocytes, and hematopoietic cells, which activate pathways leading to new functional adipose tissue and vessels.

Platelet-rich plasma (PRP) is another approach to enhance fat graft survival and aesthetic outcomes. PRP, a concentrate of plasma protein containing cytokines and angiogenic growth factors, promotes and corrects breast [6]. PRP has been extensively utilized across various medical disciplines, including orthopedics, dermatology, aesthetic medicine, and surgery [7]. Its preparation involves collecting the patient’s blood, centrifuging to separate and concentrate platelets, then injecting the platelet-rich concentrate into the target tissue for healing and regeneration [8]. PRP fat graft can be injected into the breast improving fat graft survival.

A clear distinction between reconstructive and regenerative approaches is essential to understand the goals and mechanisms of each method in breast reconstruction. Reconstructive procedures primarily focus on restoring anatomical and functional aspects of the breast post-surgery. Conversely, regenerative techniques, like CAL, rely on cellular therapies, including ADSCs and PRP to promote tissue regeneration, neovascularization, and inflammation reduction. By differentiating these approaches, this review emphasizes the distinct applications and outcomes of these innovative strategies.

This study is a literature review, focusing on the efficacy and safety of stem cells and growth factors in breast augmentation.

## Material and Methods

This narrative review was conducted to synthesize the existing literature on the role of stem-cell-assisted lipotransfer and platelet-rich plasma in breast regeneration. While we followed a structured approach to literature search, this review was not conducted according to PRISMA guidelines, as it is not a systematic review or meta-analysis.

### Search Strategy

This study included articles published from 2000 to 2024. Relevant articles were identified through searches in PubMed. To ensure comprehensive results, keywords such as “fat transplantation,” “fat graft,” “lipotransfer,” “autologous fat,” “lipofilling,” “SVF,” “Stromal Vascular Fraction,” “stem cell,” “ADSC,” “Adipose Tissue-Derived Stem Cells,” “Breast,” “Platelet-Rich-Plasma,” “PRP,” and “Breast radiation” were searched in PubMed. Boolean operators (AND, OR) were used to combine our search terms. Additionally, references cited in the retrieved articles were examined to identify further relevant studies.

### Inclusion and Exclusion Criteria

The inclusion criteria for this study include prospective, retrospective studies, systematic reviews, meta-analyses, and clinical trials published from 2000 to 2024. The exclusion criteria encompass (1) case reports and (2) non-related studies or duplicates.

### Study Selection

The search process was conducted by two researchers. Non-related articles were excluded by reviewing the title and the abstract of each study. Afterward, full-text articles were reviewed and all articles with stem-cell-assisted lipotransfer and PRP-lipotransfer were included in the present study. The authors combined and analyzed the results of the studies with emphasis on clinical trials, meta-analyses, and systematic reviews. We reinforced the existing literature with useful conclusions about the effectiveness and safety of adipose-derived stem cells and PRP in breast fat grafting, and we strengthened the evidence-based decisions.

## Results

### The Role of SVF and ADSCs in Breast Fat Grafting

Autologous fat transfer (AFT) is a widely used surgical method for breast reconstruction in plastic surgery, developed in the mid-1980 s. It is primarily indicated for correcting breast defects and reconstruction. Despite its benefits, AFT has complications such as infections, fat necrosis, cyst formation, and fat resorption. Over the past 20 years, new techniques have emerged to enhance fat graft survival.

The extraction of adipose-derived stem cells (ADSCs) was anticipated to be an innovative technique to overcome the obstacles of AFT. Cell-assisted lipotransfer (CAL) is a novel approach proposed by Matsumoto et al. in 2006, in which ADSC cells are used in combination with lipoinjection [9]. These cells are harvested from adipose tissue and isolated through enzymatic digestion, filtration, and centrifugation. ADSCs produce chemokines, cytokines, and growth factors promoting tissue neovascularization, adipogenesis, regeneration, and reduced inflammation [10].

The RESTORE-2 trial was the first clinical study to use ADSC-enriched fat grafting for breast defects after conserving surgery. It showed significant improvement in breast contouring, stable for 12 months after one or two sessions [11]. Another study with 40 patients undergoing cosmetic breast regeneration reported that CAL is effective for breast augmentation and superior to conventional lipotransfer [12]. A randomized trial demonstrated higher retention rates with ADSCs (80.2%) compared to autologous fat grafts (45.1%) [13]. Some more studies confirmed that fat graft retention in the CAL group was higher than in the conventional group [14–17].

SVF significantly enhances fat graft retention due to the regenerative properties of ADSCs [18, 19]. A clinical trial showed that SVF enrichment improved residual fat volume at 6 months [20]. Some studies showed higher fat graft survival with ADSC-enriched grafts, although others found no significant difference between SVF-enhanced and conventional grafts [21, 23]. Due to the heterogeneity of studies and the lack of long-term follow-up, randomized clinical trials are required to prove the superiority of the CAL technique over conventional lipotransfer [24].

All studies conducted regarding the role of SVF and ADSCs in breast lipofilling are described in Table 1.

**Table 1:** Studies on the role of SVF and ADSCs in breast lipofilling

Study	Publication year	Type of study	Key points
RESTORE-2 (Eur J Surg Oncol.) [11]	2012	Prospective Study	Improvement of fat graft survival with ADSCs
Yoshimura et al. (Aesthetic Plast Surg.) [12]	2008	Prospective study	No significant changes in breast volume after liposuction
Kolle et al. (Stem Cells Transl Med.) [13]	2012	Randomized clinical trial	Higher retention rate of fat with ADSCs
Dani Lutfi et al. 2020 (J Plast Reconstr Aesthet Surg.) [14]	2020	Systematic Review	Increased retention rate in the CAL group
Tissiani et al. (Stem Cells Int.) [15]	2016	Prospective Study	Higher fat graft retention in the CAL group
Jeon et al. (Aesthetic Plast Surg.) [16]	2021	Prospective Study	Fat graft retention in the CAL group was higher
Chen et al. (Cell Transplant) [17]	2021	Systematic Review and Meta-analysis	Fat graft retention in the CAL group was higher
Wei Cai et al. (Ann Plast Surg.) [18]	2018	Systematic Review	Favorable effects of SVF on fat graft survival
Gentile et al. (Int J Mol Sci.) [19]	2020	Systematic Review	Increased concentration of ADSCs in the SVF-enhanced group
Roshdy et al. (J Plast Reconstr Aesthet Surg.) [20]	2022	Randomized clinical trial	Significant improvement in residual volume at 6 months with SVF.
Karam et al. (Aesthetic Plast Surg.) [21]	2023	Systematic review and Meta-analysis	Remarkably higher fat graft survival in ADSCs-enriched fat
Shuchun Hu et al. (J Plast Reconstr Aesthetic Surg.) [22]	2024	Systematic Review and Meta-analysis	Remarkably higher fat graft survival in ADSCs-enriched fat
Ming Li et al. (Aesthetic Plast Surg.) [23]	2021	Systematic Review and Meta-analysis	Remarkably higher fat graft survival in ADSCs-enriched fat
Arshad et al. (JPRAS Open) [24]	2016	Systematic review	More randomized trials required to demonstrate the superiority of the CAL technique

### The Role of Stem Cells in Irradiated Breast Cancer Patients

Approximately two-thirds of breast cancer patients receive radiotherapy, primarily following surgery. Radiotherapy is strongly recommended for high-risk patients after mastectomy or breast-conserving procedures, as it significantly reduces recurrence rates. However, irradiated breast tissues present unique challenges for reconstructive surgery due to complications related to wound healing, fibrosis, and poor vascularization.

ADSCs have been shown to improve fat graft survival through angiogenesis and promote functional recovery of irradiated tissues [25]. Some studies highlight the potential of ADSCs and stromal vascular fraction (SVF) in tissue regeneration and wound healing after radiotherapy [26, 27]. Other studies suggest that while radiation exposure impairs the regenerative capacity of ADSCs, they retain partial functionality, making them a promising option for regenerative applications in cancer patients [28].

Furthermore, ADSCs derived from breast tissue demonstrate resistance to low-dose radiation, maintaining their proliferation and differentiation capabilities. However, high-dose radiation significantly impairs their functionality, affecting survival and regenerative potential [29]. Notably, ADSCs enhance the survival rates of random-pattern cutaneous flaps in radiation-damaged skin [30]. Additional research shows that SVF integrates effectively with irradiated tissues, resulting in improved structural and aesthetic outcomes [15, 16]. These regenerative properties contribute to better tissue healing, positioning ADSCs as promising candidates for therapeutic applications. Moreover, SVF from irradiated tissue performs comparably to SVF from non-irradiated, healthy donors in certain regenerative applications. These findings emphasize the advancements and potential of SVF-based approaches in the treatment and reconstruction of irradiated breast tissues [31].

While ADSCs hold promises for improving outcomes in irradiated patients, the long-term stability of their regenerative functions in such environments remains unclear [27]. Understanding the effects of radiation on the survival and functionality of ADSCs is critical for advancing their clinical applications. Future research should focus on standardizing protocols for ADSC enrichment and fat grafting techniques to enhance reproducibility and comparability across studies. Additionally, assessing the long-term safety and efficacy of ADSC-based treatments in irradiated environments will be crucial to their widespread adoption.

### The Role of PRP in Breast Lipofilling

Platelet-rich plasma (PRP) is a concentration of platelet-rich plasma protein derived from whole blood, which is centrifuged to remove red blood cells. Rich in platelets, growth factors, cytokines, and chemokines, PRP enhances angiogenesis, improves fat graft survival, and facilitates wound healing [32]. Multiple studies recommend adding PRP to lipofilling to enhance fat graft retention and efficiency. A randomized clinical trial demonstrated that PRP-enriched lipotransfer reduced the number of injection sessions needed for satisfactory results. Additionally, complications such as oil cysts, calcification, fat necrosis, and hematoma formation were significantly lower in the PRP group [33]. Furthermore, PRP-enhanced fat grafting exhibited decreased reabsorption rates after 3 and 6 months compared to lipofilling without PRP [33]. Another study concluded that PRP glue effectively reduces complications like seroma and hematoma, particularly in breast reduction surgeries. However, it does not significantly improve wound-healing quality [34]. Other research emphasizes the effectiveness of PRP in enhancing fat graft survival and improving both aesthetic and functional outcomes in surgical procedures. For instance, Gentile et al. [35] demonstrated that combining enhanced stromal vascular fraction with PRP contributes to fat graft maintenance in breast reconstruction. Pires Fraga et al. [36], through studies on rabbits, showed increased survival rates of free fat grafts due to the regenerative effects of PRP. Lastly, Xiong et al. [37] and Salgarello et al. [38] highlighted the synergistic effects of PRP and adipose-derived stem cells in promoting neovascularization and improving fat graft survival. Conversely, Por et al. [39] concluded that PRP had no significant effect on improving the survival of free fat grafts in their nude mouse model, raising questions about its universal applicability.

All studies conducted regarding the role of PRP in breast regeneration are described in Table 2.

**Table 2:** Studies on the role of PRP in breast lipofilling

Study	Year	Type of study		Key points
Shaaban et al. 2024 (Breast Dis.) [33]	2024	Randomized clinical trial		PRP increases fat graft survival and decreases the complication rate
Barbara Hersant et al. (Plast Reconstr Surg Glob Open) [34]	2017	Prospective study		No improvement in the wound-healing process after PRP
Gentile et al. (Stem Cells Transl Med.) [35]	2012		Clinical trial	PRP and lipofilling increase breast volume
Pires Fraga et al. (J Plast Reconstr Aesthet Surg.) [36]	2010		Randomized clinical trial	Higher fat graft survival after PRP injection
Bing-Jun Xiong et al. (Aesthetic Plast Surg.) [37]	2018		Comparative study	Higher fat graft survival after PRP injection
Por YC et al. (J Plast Reconstr Aesthet Surg.) [38]	2008		Prospective study	PRP-enriched fat graft does not improve the aesthetic result
Salgarello et al. (Plast Reconstr Surg.) [39]	2011	Controlled clinical trial		No significant clinical outcomes after the use of PRP in fat graft

### Complication Rate with CAL Technique

The aim is to review the literature on complications associated with ADSCs or SVF-based lipofilling and compare it with conventional fat grafting. A prospective study supported the safety of SVF-enriched lipofilling due to the low rates of complications, with similar fat necrosis occurrence in both SVF and autologous lipofilling groups [16]. Other studies reported no significant difference in complication rates between the CAL and conventional lipotransfer groups [13, 23], except for injection site cysts in 14.9% of cases [11], small cystic formations [12, 40], and calcification in 83% of cases compared with the conventional group [24]. Some authors [17, 35] report higher complication rates with the CAL technique when compared to AFT injections [41].

The variance in complication rates of CAL is likely due to the non-standardized technique, with infection and hematoma formation being the most common complications. The short-term complication rate was similar between the implant-based reconstruction group and the implant with lipofilling group [42].

### SVF, ADSCs, and Risk of Oncogenesis

Autologous fat transfer has become a cornerstone in the field of plastic surgery, particularly for breast reconstruction. Despite its widespread use, AFT is not without complications, prompting ongoing research into enhancing its efficacy and safety. Limited evidence from in vivo studies exists concerning the safety of breast fat grafting [43]. The oncological safety of ADSCs remains unclear at present [44, 45].

According to some studies, autologous fat grafting does not increase the recurrence risk, although ADSCs should be used with caution in patients with a history of breast cancer surgery [46]. The CAL technique showed no increase in recurrence rate compared to controls [11, 47–49], with no loco-regional or metastatic disease observed post-radiation [15, 16].

While the aforementioned clinical studies conclude that adipose fat transposition does not cause significant loco-regional recurrence, other experimental and clinical studies support the opposite [51, 53–57]. ADSCs and extracellular vesicles (EVs) demonstrate antitumor activity in mouse models, but increased recurrence risk has been shown in certain cases [50, 51]. EVs enhance tissue regeneration and modulate immune responses, but their role in oncogenesis is still uncertain [52]. Various growth factors and pathways linked to ADSCs may contribute to the tumor microenvironment and tumorigenesis. Specifically, various growth factors and molecular pathways, such as TGF-β1/Smad, PI3 K/AKT, adipsin, CXCL1/8, CK 18+19, and interleukin-6, potentially contribute to the tumor microenvironment and tumorigenesis in breast cancer cells [53–57]. Further prospective studies and long-term follow-up are needed to prove the oncological safety of this procedure [58, 59]. All studies conducted regarding the oncological safety of ADSCs are summarized in Table 3.

**Table 3:** Studies concerning the oncological safety of ADSCs in breast

Study	Publication year	Type of study	Key points
Perez-Cano et al. (Eur J Surg Oncol) [11]	2012	Prospective study	CAL did not increase the recurrence rate compared to the control group
Tissiani et al. (Stem Cells Int.) [15]	2015	Prospective Study	No loco-regional or metastatic disease observed in the CAL group after radiation therapy
Jeon et al. (Aesthetic Plast Surg.) [16]	2021	Prospective Study	No loco-regional or metastatic disease observed in the CAL group after radiation therapy
Valente et al. (Transl Breast Cancer Res.) [43]	2024	Systematic review	Limited evidence regarding the safety of breast fat graft
Claro et al. (Br J Surg.) [44]	2012	Systematic Review	Oncological safety of ADSCs remains unclear
Gutowski et al. (Plast Reconstr Surg.) [45]	2009	Practice Guideline	Oncological safety of ADSCs remains unclear
Kai Wang et al. (Aesthetic Plast Surg.) [46]	2023	Meta-analysis	ADSCs should be used with caution in patients with breast cancer surgery
Mazur et al. (Adv Clin Exp Med.) [47]	2018	Prospective Study	CAL did not increase the recurrence rate compared to control group
Calabrese et al. (Eur Rev Med Pharmacol Sci.) [48]	2018	Prospective Study	CAL did not increase the recurrence rate compared to control group
Gentile et al. (J Clin Med.) [49]	2019	Prospective Study	CAL did not increase the recurrence rate compared to control group
Li et al. (Pharmacol Res.) [50]	2020	Prospective Study	EVs demonstrate antitumor activities in mouse models of breast cancer
Kamat et al. (Plast Reconstr Surg.) [51]	2015	Prospective Study	Increased recurrence risk in cases of intraepithelial neoplasms
Wu S et al. (Mol Med Rep.) [53]	2018	Prospective Study	Signaling pathways promote the tumor microenvironment of breast cancer cells
Goto et al. (Oncogene) [54]	2019	Prospective Study	The role of adipsin in tumor development
Wang Y et al. (Stem Cells) [55]	2017	Prospective Study	CXCL1/8 secreted by ADSCs may enhance breast cancer angiogenesis
Yang J et al. (J Cell Mol Med.) [56]	2015	Prospective Study	CK 18+19 in ADSCs raise concerns about oncological safety
Wei HJ et al. (Oncotarget) [57]	2015	Prospective Study	JAK2/STAT3 pathway may promote tumor angiogenesis
Waked et al. (Breast) [58]	2017	Systematic Review	Concerns over the oncologic safety remain controversial
Charvet et al. (Ann Plast Surg.) [59]	2015	Systematic Review	Oncological concerns have risen around the safety of adipose fat transfer

### PRP and Risk of Oncogenesis

PRP has been explored for its potential benefits in surgical procedures, particularly in breast regeneration following mastectomy or lumpectomy. PRP aims to improve wound healing, reduce complications, and enhance overall surgical outcomes. However, its oncological safety remains a concern due to growth factors that could theoretically promote tumor growth and recurrence [60].

The results on PRP’s oncological safety in breast cancer patients are conflicting. Some studies suggest that PRP can be safely used without increasing the risk of cancer recurrence. Specifically, a study evaluating the application of PRP in sentinel lymph node biopsy procedures for breast cancer found no increase in local recurrence or distant metastases within a 30-month follow-up period [61]. Another study reported no cancer recurrence when PRP was applied to venous port sites after removal in high-risk cancer patients [62].

Conversely, concerns have been raised about PRP promoting tumor growth due to growth factors such as platelet-derived growth factor (PDGF), vascular endothelial growth factor (VEGF), epidermal growth factor receptor (EGFR or HER), and transforming growth factor-beta (TGF-β). These certain growth factors facilitate processes that allow tumor cells to survive and proliferate [63, 64].

Despite the concerns about the oncological safety of PRP, the hypothesis that PRP could lead to malignant transformation has not been proven. More studies are required to investigate the safety of PRP in previous areas of cancer excision. Until more conclusive evidence is obtained, the use of PRP in breast regeneration for cancer patients should be approached with caution.

## Discussion

This study aimed to evaluate the efficacy and safety of stem cells and growth factors in breast augmentation. The widespread application of stem cells and growth factors in breast deficit repair have opened new avenues in plastic surgery. Cell-assisted lipotransfer (CAL), which enriches autologous fat grafts with adipose-derived stem cells (ADSCs), improves fat graft survival. However, high-level evidence of efficacy and safety is lacking, with concerns about SVF cells promoting tumor growth and metastasis. Kamat et al. [51] reported that ADSCs might promote breast cancer progression and metastatic spread. Conversely, other studies have concluded that CAL does not increase the risk of cancer recurrence. For instance, Wang et al. [55] found no significant increase in cancer recurrence rates after autologous fat graft.

Previous irradiation in the breast can affect tissue, with mixed findings on how it impacts fat graft survival [62]. Several studies concluded that the growth of adipose-derived stem cells (ADSCs) is unaffected by radiation, improving fat graft retention, whereas others note limited efficacy of the CAL technique in irradiated breasts. More research is needed to prove the efficacy and oncological safety of adipose-derived cells in irradiated breasts.

PRP has gained an important role in aesthetic and regenerative medicine, improving fat graft survival, angiogenesis, and wound healing. However, data on PRP’s benefits in breast fat graft retention are inconsistent, with some studies suggesting benefits and others finding no significant differences.

### Study Limitations and Future Research Directions

In breast reconstruction, particularly after radiation-induced tissue damage, the development and application of regenerative techniques such as ADSCs and PRP hold significant promise. The safety of ADSC and PRP use, particularly in irradiated tissues, remains an area of concern. Research must evaluate oncological safety and recurrence risks, alongside the long-term effectiveness of these regenerative techniques.

The variability in patient selection criteria is a recurring challenge in studies involving ADSC- and PRP-enriched grafts for breast reconstruction. Current research often includes diverse patient populations, varying in factors such as tissue quality, radiation exposure, and overall health status [26]. This lack of uniform criteria can lead to inconsistent findings, as patient-specific characteristics significantly influence outcomes such as graft survival and tissue regeneration. By doing so, future trials should involve more homogeneous populations, ensuring reliable and comparable results.

Additionally, procedural variability, such as differences in SVF isolation, PRP concentration, and graft preparation methods, complicates the ability to draw robust conclusions from existing studies [65]. Without standardized protocols, it becomes difficult to assess the true efficacy of these regenerative techniques or compare results across studies reliably. Achieving reliable outcomes in regenerative breast reconstruction relies on minimizing variability in patient selection criteria and procedural methods.

The optimal placement of ADSC- or PRP-enriched grafts is critical in determining the efficacy of regenerative breast reconstruction. Placement within the breast tissue may enhance tissue integration, promoting vascularization and improving regenerative outcomes, particularly in cases involving irradiated tissues. Conversely, positioning the grafts in supra- or subglandular layers can offer better structural support, especially for patients requiring volume restoration in conjunction with implants or flaps. Despite these theoretical advantages, current studies remain inconclusive, highlighting the need for systematic research to compare outcomes. Standardized protocols and randomized trials are essential to establish evidence-based guidelines for optimal placement strategies [66].

A consistent challenge in the field is the absence of objective outcome measures, which undermines the comparability of results. To address this, future research must adopt clearly defined and standardized metrics for assessing fat graft survival, tissue regeneration, and patient satisfaction. Quantitative imaging tools and validated patient-reported outcome measures should be central to this effort [67].

Equally important is the need to understand the molecular mechanisms by which SVF and ADSCs enhance fat graft survival and tissue repair. These mechanisms, including the promotion of neovascularization and the modulation of inflammation, are critical to optimizing clinical applications and unlocking the full potential of regenerative medicine [68].

PRP concentration is another key area for optimization. Determining the ideal PRP concentration and understanding its role in graft survival and tissue rejuvenation is essential. Research on the synergistic effects of PRP and ADSCs could lead to improved outcomes in breast reconstruction [69].

By addressing these challenges, future clinical studies must focus on adopting standardized protocols, ensuring large sample sizes, and conducting long-term follow-ups. This will enable robust, reproducible results, establishing CAL, ADSC, and PRP applications as transformative tools in breast reconstruction. These innovations have the potential not only to improve aesthetic and functional outcomes but also to significantly enhance the quality of life for patients undergoing breast reconstruction.

## Conclusion

This review aimed to evaluate the efficacy and safety of ADSCs and PRP-enriched fat grafting. These techniques have entered clinical practice for aesthetic breast regeneration, as they are innovative and based on autologous tissue. However, the use of autologous fat is associated with high absorption rates. Research has focused on the use of growth factors and stem cells to increase neovascularization and fat survival. The existing literature suggests that the use of stem cells and growth factors may be safe and efficient, giving promise to significantly enhance the quality of life for patients undergoing breast reconstruction. While the preliminary results are promising, the journey toward fully integrating stem cells and PRP in breast augmentation is still ongoing. The collaboration between researchers, clinicians, and regulatory bodies will be crucial in overcoming the current challenges and unlocking the full potential of these advanced therapies.
